# From data to practice: brain meningioma treatment in elderly patients – a survey of the Italian Society of Neurosurgery (SINch®) and systematic review and meta-analysis

**DOI:** 10.1007/s10143-024-02524-8

**Published:** 2024-07-31

**Authors:** Tamara Ius, Giovanni Raffa, Denis Aiudi, Pier Paolo Panciani, Giuseppe Maria Della Pepa, Federico Pessina, Domenico Solari, Teresa Somma, Filippo Flavio Angileri, Michele Nichelatti, Paolo Cappabianca, Vincenzo Esposito, Maurizio Fornari, Domenico Gerardo Iacopino, Alessandro Olivi, Francesco Sala, Luigi Maria Cavallo, Alessandro La Camera, SINch NeuroOncology Study Group, Giuseppe Barbagallo, Andrea Barbanera, Giacomo Beggio, Silvio Domenico Bellocchi, Claudio Bernucci, Manuela Anna Caroli, Marco Cenzato, Alessandro Della Puppa, Andrea Di Cristofori, Riccardo Draghi, Antonio Fioravanti, Marco Maria Fontanella, Alessandro Frati, Filippo Gagliardi, Diego Garbossa, Antonino Germanò, Maurizio Iacoangeli, Luigi Antonio Lattanzi, Federico Legnani, Davide Locatelli, Vincenza Maiola, Nicola Montemurro, Giovanni Muscas, Antonio Nicolato, Sergio Paolini, Giacomo Pavesi, Maurizio Piparo, Domenico Policicchio, Angelo Pompucci, Antonino Raco, Marta Rossetto, Giovanni Sabatino, Antonio Santoro, Silvio Sarubbo, Alba Scerrati, Francesco Signorelli, Fabio Spanu, Giannantonio Spena, Roberto Stefini, Stefano Telera, Luigino Tosatto, Roberto Trignani, Silvana Tumbiolo, Francesco Volpin, Giampaolo Zambon, Donato Carlo Zotta

**Affiliations:** 1 Neurosurgery Unit, Head-Neck and Neurosciences Department, Santa Maria Della Misericordia University Hospital, Udine, Italy; 2 Neurosurgery Unit, Department of Biomedical and Dental Sciences and Morphofunctional Imaging, University of Messina, Messina, Italy; 3 Neurosurgical Department, Università Politecnica Delle Marche, Marche General University Hospital, Ancona, Italy; 4 Present Address: Neurosurgery Unit, Department of Medical and Surgical Specialties, Radiological Sciences and Public Health, University of Brescia, Spedali Civili Di Brescia, Brescia, Italy; 5 Institute of Neurosurgery, IRCCS Fondazione Policlinico Universitario Agostino Gemelli, Catholic University, Rome, Italy; 6 Department of Biomedical Sciences, Humanitas University, Pieve Emanuele, Milan, Italy; 7 Division of Neurosurgery, Department of Neurological Sciences, Università Degli Studi Di Napoli Federico II, Naples, Italy; 8 Department of Clinical Research & Innovation, Grande Ospedale Metropolitano Niguarda, Milan, Italy; 9 Department of Neurosurgery, IRCCS Neuromed, Pozzilli, Italy; 10 Neurosurgery Department, IRCCS Humanitas Research Hospital, Milan, Italy; 11 Department of Biomedicine Neurosciences and Advanced Diagnostics, Neurosurgical Clinic, AOUP “Paolo Giaccone”, Post Graduate Residency Program in Neurologic Surgery, School of Medicine, University of Palermo, Palermo, Italy; 12 Department of Neurosciences, Biomedicine and Movement Sciences, Neurosurgery Unit, University of Verona, Verona, Italy; 13 Division of Neurosurgery, Department of Neuroscience, Grande Ospedale Metropolitano Niguarda, Milano, Italy; 14 Department of Neurosurgery, Policlinico “G. Rodolico” University Hospital, Catania, Italy; 15 Neurosurgery Unit, SS. Antonio and Biagio and Cesare Arrigo Hospital Alessandria, Alessandria, Italy; 16 Department of Neurosurgery, San Bortolo Hospital, Vicenza, Italy; 17 Unit of Neurosurgery, Sant’Anna Hospital, Como, Italy; 18 Division of Neurological Surgery, ASST Papa Giovanni XXIII, Bergamo, Italy; 19 Neurosurgery Unit, Department of Surgery, Fondazione IRCCS Ca’ Granda Ospedale Maggiore Policlinico, University of Milan, Milan, Italy; 20 Neurosurgery Department, Padua Hospital, Padua, Italy; 21 Neurosurgery Unit, Department of Neuroscience, San Gerardo Hospital, Monza, Italy; 22 Department of Neurosurgery, Maria Cecilia Hospital, GVM Care&Research, Cotignola (RA), Italy; 23 Medical Oncology and Translational Research Unit, Cremona Hospital, Cremona, Italy; 24 Neurosurgery Division, Human Neurosciences Department, Sapienza University, Rome, Italy; 25 Department of Neurosurgery and Gamma Knife Radiosurgery, San Raffaele University and IRCCS San Raffaele Scientific Institute, Milan, Italy; 26 Department of Neuroscience Rita Levi Montalcini, Neurosurgery Unit, University of Turin, Turin, Italy; 27 Department of Neurosurgery, Anthea Hospital, GVM Care&Research, Bari, Italy; 28 Department of Neurosurgery, National Neurologic Institute IRCCS C. Besta, Milan, Italy; 29 Neurosurgical Clinic, Insubria University, ASST Settelaghi, Varese, Italy; 30 Department of Neurosurgery, Azienda Ospedaliero Universitaria Pisana, Pisa, Italy; 31 Neurosurgery Unit, Department of Neuroscience, Psychology, Pharmacology and Child Health, University of Florence, Florence, Italy; 32 Department of Neuroscience, University Hospital, AOUI, Verona, Italy; 33 Department of Neurosurgery, Sant’Agostino Estense Hospital, Modena, Italy; 34 Department of Neurosurgery, Azienda Ospedaliera Universitaria di Sassari, Sassari, Italy; 35 Department of Neurosurgery, S. Maria Goretti Hospital, Latina, Italy; 36 Division of Neurosurgery, Department of NESMOS, AOU Sant’Andrea, Sapienza University, Rome, Italy; 37 Department of Neurology and Psychiatry, Neurosurgery, “Sapienza” University of Rome, Rome, Italy; 38 Department of Neurosurgery, Santa Chiara Hospital, Trento, Italy; 39 Department of Neurosurgery, Sant’Anna University Hospital, Ferrara, Italy; 40 Department of Basic Medical Sciences, Neurosciences and Sense Organs, University of Bari, Bari, Italy; 41 Department of Neurosurgery, Santa Corona Hospital, Pietra Ligure (SV), Italy; 42 Neurosurgery Unit, Fondazione IRCCS Policlinico S. Matteo, Pavia, Italy; 43 Departiment of Neurosurgery, Legnano Hospital, Legnano (MI), Italy; 44 UOSD Neurosurgery, IRCSS National Cancer Institute “Regina Elena”, Rome, Italy; 45 Department of Neurosurgery, Ospedale M. Bufalini, Cesena, Italy; 46 Department of Hospital Neurosurgery, AOU Ospedali Riuniti, Ancona, Italy; 47 Division of Neurosurgery, Villa Sofia Hospital, Palermo, Italy; 48 Neurosurgical Unit, Ospedale Spirito Santo, Pescara, Italy; 49 Fabrizio Spaziani Hospital, Frosinone, Italy

**Keywords:** Meningioma, Elderly, Surgery, Radiosurgery, Aging, Outcome, Morbidity, Mortality

## Abstract

The management of meningioma in elderly patients (MEP) presents a complex and evolving challenge. Data available offer conflicting information on treatment options and complications. This survey aimed to examine the current approach to MEP, comparing the national profile to data in the current literature. A survey addressing the treatments options and management of meningioma in elderly was designed on behalf of SINch**®** (Società Italiana di Neurochirurgia) and sent via email to all Chiefs of Neurosurgical Departments. The survey remained open for responses from May 5th, 2022, until November 21st, 2022. A search of the literature published between January 2000 and March 2023, in accordance to PRISMA guidelines, was included. A total of 51 Neurosurgical centers participated in the survey. The caseload profile of each center influences the choice of treatment selection (Stereotactic Radiosurgery *versus* open surgery) (*p* = 0.01) and the consolidated practice of discussing cases within a multidisciplinary group (*p* = 0.02). The pooled meta-analysis demonstrated a significant increased risk in the elderly group for permanent deficits (*p* < 0.00001), postoperative infections (*p* = 0.0004) and hemorrhage (*p* = 0.0001), perioperative mortality (*p* < 0.00001), and medical complications (*p* < 0.00001) as compared to the young population. This study presents the initial comprehensive analysis of the existing trends in the surgical management of MEP in Italy. The significant variation in practices primarily stems from the absence of standardized guidelines. While most centers have adopted an integrated approach, there is a need to promote a multidisciplinary care model. Prospective studies are needed to gather robust evidence in this clinical setting.

**Supplementary Information:**

The online version contains supplementary material available at 10.1007/s10143-024-02524-8.

## Introduction

Meningiomas account for 16–36% of all intracranial tumors in adults. Their incidence is about 1 case per 12,500 individuals in the general population but increases significantly with age, reaching 1 case per 2,000 individuals in those over 80 years old [1]. Enhanced life expectancy and better diagnostic technology have resulted in more cases of symptomatic and incidental intracranial meningiomas, this suggests a growing demand for neurosurgical treatment, particularly in older patients, and raises questions about the best risk–benefit balance among available treatment options [2, 3].

Traditionally, surgical resection remains the gold standard of treatment for large, symptomatic lesions, and rapidly growing tumors under surveillance [3, 4].

The primary goal of surgical treatment is to maximize tumor removal while minimizing morbidity and mortality. In meningioma elderly patients (MEP) existing literature has demonstrated a broad spectrum of mortality (ranging from 0 to 45%) and complication rates (varying from 10 to 39%) [5–22]. Furthermore, several comparative studies have indicated that surgery in MEP carries a higher risk of mortality and morbidity when compared to younger patients [5–7].

The shorter life expectancy of older patients and higher comorbidities ratio has thus led to consideration of subtotal resection (STR) as an alternative to gross-total resection (GTR), often combined with adjuvant radiotherapy (RT) or stereotactic radiosurgery (SRS), especially in skull-base meningioma (SBM) [3–5, 14, 19, 20, 23, 24].

In addition, the ‘wait and see’ strategy, especially in patients with comorbidities and minimally symptomatic tumors, might be preferred. Nonetheless, it was reported that elderly patients who opted for conservative treatment exhibited higher tumor-related mortality rates in comparison to those who underwent surgical resection. Furthermore, elderly patients appear to experience a higher occurrence of atypical and anaplastic meningiomas, which exhibit more aggressive behavior compared to benign meningiomas and have the potential to complicate the clinical progression [3, 4, 24].

A consensus on the surgical approach for MEP has not yet been reached, and there is limited establishment of predictive factors for this patient group who undergo surgical resection [3, 8]. This leads to the issue of whether the initial surgical intervention is equally effective for older patients as it is for younger adults, and whether the postoperative clinical outcomes in terms of complications, surgical morbidity, and mortality are comparable between these two age groups.

This investigation aims to elucidate the existing evidence and tackle the recognized challenges linked to MEP, discussing the current clinical mindset of Italian neurosurgeons in light of recent literature data.

## Materials and methods

### Survey study design and targeted population

A survey addressing the MEP treatments options was designed by the Coordinators of Neuroncology (T.I.), Stereotactic Radiosurgery (A.L.), end Neuroendoscopy Section (L.M.C.) of SINch, using an online tool (Survey Monkey© Inc., San Mateo, California, USA, www.surveymonkey.com). The SINch members Board gave their approval to the survey, which was subsequently sent via email to all Chiefs of Neurosurgical Department requesting a single referent for each center. The survey remained open from May 5th, 2022, until November 21st, 2022. Data were collected anonymously. The survey included 31 queries summarized in Table 1, exploring four domains: (1) demographics and other respondents’ characteristics; (2) elderly definition and risk scales; (3) questions on treatment options (4) questions on perioperative and postoperative management. Completion of the entire survey took around 8–10 min.

**Table 1:** Summary of Results of Italian survey about surgical management of MEP, stratified according the investigated domains

Demographics and other respondent characteristics		
Query	Response	*N* (%)
Indicate the type of hospital that you work for	Academic Hospital	17 (33.3)
	Public Hospital	35 (66,7)
Indicate your current working position	Chief	21 (41.2)
	Full-time Professor	8 (15.6)
	Senior Researcher	3 (5.8)
	Hospital Clinician	19 (37.5)
What is your level of experience in the surgical management of cranial meningiomas?	< 10 years	10 (19.6)
	10–15 years	7 (13.7)
	> 15 years	34 (66.7)
What is your age group?	35–45	19 (37.3)
	46–55	13 (25.4)
	> 55	19 (37.3)
Gender	Male	45 (88.2)
	Female	6 (11.8)
In which region do you work?	North	29 (56.9)
	Center	14 (27.5)
	South	8 (15.6)
How many patients diagnosed with meningioma are treated on average each year at your center?	< 50 cases/year	14 (27.5)
	50–99 cases/year	25 (49)
	> = 100 cases/year	12 (23.5)
What treatments are currently available in your Center? (*multiple choice available*)	Endoscopic surgery	36 (70.6)
	Stereotactic radiosurgery	28 (55.9)
	Fractionated stereotactic radiotherapy	38 (74.5)
Which preoperative MRI protocol do you use?	Standard MRI	6 (11.8)
	Advanced imaging protocol	45 (88.2)
Select the intraoperative tools available in your Department (*multiple choice available*)	Neuronavigation	51 (100)
	Electrophysiological monitoring/stimulation	46 (90.2)
	Intraoperative ultrasound	31 (60.8)
	5ALA (to evaluate the bone infiltration)	14 (27.5)
	Intraoperative CT	10 (19.6)
	Intraoperative laser	6 (11.8)
Elderly definition and risk scales		
Query	Response	*N* (%)
What is the percentage of elderly patients do you operate in a year?	10–20 %	8 (15.7)
	20–50%	39 (76.5)
	> 50%	4 (7.8)
How do you define a patient as being elderly?	> 70 years	34 (66.7)
	> 75 years	15 (29.4)
	> 80 years	2 (3.9)
Which grading system do you use to assess the preoperative risk in elderly patients) (*multiple choice available*)	ASA	51 (100)
	CCI	7 (13.7)
	ECOG	10 (19.6)
	Other: CRGS and Clinical Frailty Scale	2 (3.9)
Do you discuss cases within a multidisciplinary group for the stratification of the surgical risk and definition of the best therapeutic option (Surgery versus SRS, or debulking and subsequent SRS)?	Always	19 (37.3)
	Never	5 (9.8)
	In selected cases	27 (52.9)
	- midline lesions < 3 cm maximum diameter	- 14 (51.8)
	- high comorbidity risk	- 5 (18.6)
	- both	- 8 (29.6)
Does the factor of age have an influence on the surgical indication?	Only for those cases with high comorbidity preoperative risk	39 (76.4)
	Always	11 (21.5)
	Never	1 (1.9)
Questions on treatment options		
Query	Response	*N* (%)
Does the factor of age play a role when deciding between an endoscopic approach compared to the classic microsurgical one?	Always	3 (5.9)
	Never	38 (74.5)
	In selected cases	10 (19.6)
For which meningiomas do you select the endoscopic approach as the first option? (*open question*)	Midline lesions with a maximum diameter < 3 cm	39 (76.4)
	For those cases with high comorbidity preoperative risk	20 (39.2)
	Residual management	34 (66.7)
In what percentage of median skull base meningiomas (tuberculum, planum, Clivus) is the endoscopic procedure the first choice? (*open question*)	< 10%	28 (54.8)
	11–30%	17 (33.3)
	> 31%	6 (11.8)
Does the factor of age influence the choice of a SRS approach compared to "open" surgery?	Always	4 (7.8)
	Never	15 (29.4)
	In selected cases	32 (62.8)
For which meningiomas do you select the neuroradiosurgical approach as the first option? (*open question*)	Midline lesions with a maximum diameter < 3 cm	39 (76.4)
	For those cases with high comorbidity preoperative risk	20 (39.2)
	Residual management	34 (66.7)
In cases of meningiomas of the convexity associated with significant hyperostosis, is the cranioplasty procedure performed during the same surgical procedure?	Always	48 (94)
	Never	3 (6)
Which material do you use for cranioplasty procedure? Responders 44/48	Custom made	13 (29.5)
	Acrylic/Alumina Ceramics/Methyl- Methacrylate	27 (61.4)
	Titanium	4 (9.1)
How do you manage elderly patients with asymptomatic incidental meningiomas without edema	Preventive SRs	0
	Preventive surgery	0
	Wait and scan	51 (100)
How do you manage elderly patients with asymptomatic incidental meningiomas showing a volumetric MRI progression	Wait and see with regular MRI follow-up	12 (23.6)
	SRs	6 (11.8)
	Surgery	33 (64.6)
Questions on perioperative and postoperative management		
Query	Response	*N* (%)
For which cases is preoperative AGF and embolization used (*open question*)	Always	1 (1.9)
	Never	8 (15.6)
	In selected cases:	42 (84.5)
	- vascular encasement	- 15 (35.7)
	- skull base lesions	- 25 (59.5)
	- giant supratentorial lesions	- 7 (16.7)
In cases of meningiomas with incomplete removal and WHO diagnosis I what is the postsurgical treatment of choice?	Wait and scat with regular MRI follow-up	42 (82.4)
	SRs	9 (17.6)
In cases of meningiomas with incomplete removal and WHO diagnosis II what is the postsurgical treatment of choice?	Wait and scan with regular MRI follow-up	17 (33.3)
	fRT/SRs	33 (64.8)
	Adrotherapy / Proton Therapy	1 (1.9)
In cases of meningiomas with incomplete removal and WHO diagnosis III what is the postsurgical treatment of choice? :	fRT/SRs	22 (43.1)
	Adrotherapy /Proton Therapy	29 (56.9)
Do you perform early (within 24 hours) DVT/TE prophylaxis in elderly patients operated for meningioma? Total responders 26/51	Yes	24 (92.4)
	No	2 (7.6)
How do you make DVT/TE prophylaxis in elderly patients operated for meningioma? (*multiple choice*) Total responders 26/51	Identifying patients at high risk for the development of venous TE	4 (15.4)
	Preoperative compression stockings	19 (73.1)
	Intraoperative intermittent pneumatic compression	8 (30.8)
	Low molecular weight heparin (LMWH) (24 hours after surgery)	24 (92.4)
	Early mobilization	24 (92.4)
	Continuous postoperative saturation monitoring for 48 h after surgery	2 (7.7)
Do you perform early brain CT scan before starting prophylaxis? Total responders 26/51	Yes	19 (73.1)
	No	7 (26.9)

### Literature review

An extensive review of published studies was conducted using the Preferred Reporting Items for Systematic Reviews and Meta-Analyses (PRISMA) guidelines.

### Review question

The review questions, according to the PRISMA statement, were formulated following the PICO (P: patients; I: intervention; C: comparison; O: outcomes) scheme, as it follows: In newly diagnosed meningioma (P), has surgery in patients age over 70 (I) revealed as effective when compared to surgery in younger ages (C), in terms of *morbidity and mortality* (O)?

### Search strategy

A specific literature search protocol was developed to collect data from studies reporting the comparison of morbidity and mortality after surgical treatment of elderly meningioma group (MEP, ≥ 65 years old) vs. young meningioma group (YMG, < 65 years old). For the most comprehensive detection of papers the search query was built as follows using a combination of medical subject headings (MeSH): “elderly” [MeSH] AND “meningioma”[MeSH] and free text terms: “surgery” OR “postoperative deficits*” OR “post-operative mortality” OR “post-operative morbidity” OR “stereotactic radiosurgery” OR “radiosurgery” OR “outcome”.

We included studies published from January 2000 to March 2023, comparing the following outcomes in MEP *versus* YMG: 1) neurological complications, 2) medical complications, 3) mortality.

Case reports, review articles, meta-analyses, abstracts, reports of aggregated data and reports on multimodal therapy where surgery was not the primary treatment were excluded. In addition, exclusion criteria encompassed language other than English, non-comparative studies, and non-reported quantitative data.

Two authors, D.A and G.R, independently reviewed paper titles and abstracts, removing duplicates. In the second review phase, they assessed papers for inclusion based on specific criteria. The references of these papers were also checked (forward search) for any missed papers. Any disagreements during the screening process were resolved through consensus, and interobserver agreement was measured using Cohen's k coefficient.

### Quality scoring

The ROBINS-I tool was applied to evaluate the Risk of Bias (RoB) in non-randomized controlled trials (non-RCTs), detected by the screening process [25].

The overall RoB was categorized as critical, serious, moderate, low, or with no available information. The RoB assessment was performed independently by two investigators (T.I. and D.A.). Seven main domains for assessing the risk of systematic errors in selected papers were included: Bias due to confounding; Bias in participant selection; Bias in intervention classification; Bias from deviations in intended interventions; Bias due to missing data; Bias in outcome measurement and Bias in result selection.

### Statistical analysis

Categorical variables are reported as absolute numbers and percentages whereas continuous variables are reported as median value ± standard deviation. The results of the survey were analyzed by using the Chi-square of Fisher’s exact tests to compare categorical variables. In particular, we analyzed any statistically significant difference about treatment strategies in elderly meningioma management among centers with different caseloads. Indeed, the results of the survey responses were originally tabulated by number of cases per center per year: high caseload (> 100 cases per year), intermediate caseload (51–99 cases per year), and low caseload centers (< 50 cases per year). Differences were analyzed comparing 1) high vs. intermediate vs. low caseload centers; 2) low + intermediate vs. high caseload centers; 3) low vs. intermediate + high caseload centers. A 2-tailed P value of 0.05 was considered statistically significant for all analyses. When HR was not reported, univariate Cox proportional hazards model was used to calculate HR and 95% confidence intervals (CI). For the meta-analysis, the raw data regarding patient’s demographic, the occurrence of any postoperative 1) neurological complication, including new deficits and seizures, 2) surgical complication including CSF leakage, hemorrhage, infection, 3) medical complication, and 4) mortality were collected in a specific database using Microsoft Excel 2019 (Microsoft Corp, Redmond, WA). The individual and pooled odds ratio (OR) for the different analyzed outcome were calculated by using the Mantel–Haenszel (M-H) fixed effect model in the elderly vs. the young populations. For each outcome, 95% confidence intervals (CIs) and 2-sided p values were calculated. A *p* < 0.05 was considered statistically significant. Heterogeneity was evaluated using the I^2^ statistic. An I^2^ value > 50% was considered indicative of significant heterogeneity. Publication biases were defined through the visual inspection of the funnel plot for each outcome. The statistics for the meta-analysis was performed using the software Cochrane Review Manager (RevMan, Version 5.4. The Nordic Cochrane Centre, The Cochrane Collaboration 2014, Copenhagen, Denmark).

## Results: Italian survey

Overall, 120 Neurosurgical Centers were electronically contacted, of whom 51(42.5% response rate) answered the questions raised by the survey. Data regarding responder demographics and areas of practice, according to the four topic domains, are summarized in Table 1.

Regarding the annual caseload, 23.5% of the Centers stated a high case-load while 49% and 27.5% had an intermediate and low caseload, respectively. In daily clinical practice, specific predictive scores for the functional outcome are routinary used only by a few Centers (17.6%). In the majority of Centers (76.4%) age per se is not a factor influencing the surgical decision. More than half of responders identified patients over 70 years of age as elderly.

The endoscopic and SRS approach resulted to be emerging options in MEP management. Differently, the *case-by-case* discussion within a multidisciplinary dedicated board, is currently still poorly practiced.

For those cases of meningiomas of the convexity associated with significant hyperostosis, in almost all Centers the cranioplasty procedure is performed during the same surgical resective-time. The choice of the material used is extremely heterogeneous.

All responders agreed about the wait and scan approach for those cases with incidental meningioma. Surgery remains the treatment of choice at the time of volumetric progression, while preoperative angiography and embolization still remains an uncommon procedure.

Overall the postoperative management options related to venous thrombo-embolism (VTE) prophylaxis and adjuvant treatments resulted consistent with data of current literature.

The adjuvant postoperative treatment selection is heterogeneous especially for WHO II meningiomas with STR.

The association between treatment strategies or choice and caseload of the responder Centers was explored (Table 2). Significant differences were mainly observed regarding the influence of the factor “age” on surgical indications and the management discussion during multidisciplinary meetings.

**Table 2:** The association between treatment strategies or choice in management of MEP and case-load of the responder Centers

QUERY	CASE LOAD		
	< 50; 50–100; > 100	< 99; > = 100	< 49; > = 50
Does the factor of age have an influence on the surgical indication?	*P* = 0.10	***P*** **= 0.02**	*P* = 0.5
Do you discuss cases within a multidisciplinary group for the stratification of the surgical risk and definition of the best therapeutic option?	*P* = 0.05	*P* = 0.16	***P*** **= 0.01**
Does the factor of age influence the choice of a SRS approach compared to "open" surgery?	*P* = 0.06	*P* = 0.4	***P*** **= 0.01**
Does the factor of age play a role when deciding between an endoscopic approach compared to the classic microsurgical one?	*P* = 0.19	*P* = 0.13	*P* = 0.16
In cases of meningiomas of the convexity associated with significant hyperostosis, is the cranioplasty procedure performed during the same surgical resective procedure?	*P* = 0.17	*P* = 0.67	*P* = 0.11
In cases of meningiomas with incomplete removal and WHO diagnosis I what is the postsurgical treatment of choice	*P* = 0.92	*P* = 0.74	*P* = 0.8
In cases of meningiomas with incomplete removal and WHO diagnosis II what is the postsurgical treatment of choice	*P* = 0.82	*P* = 0.76	*P* = 0.5
In cases of meningiomas with incomplete removal and WHO diagnosis III what is the postsurgical treatment of choice	*P* = 0.74	*P* = 0.38	*P* = 0.97

The *factor age* has a significant impact on surgical indications in Centers with high caseload, with a statistically significant difference compared to Centers with intermediate and low caseload (*p* = 0.02). Additionally, the *factor age* plays a significant role in the choice between an SRS approach and "open surgery” in Centers with high and intermediate caseload compared to those with low caseload (*p* = 0.01).

Moreover, cases are more frequently discussed within multidisciplinary teams for risk stratification and defining the best treatment option in Centers with high and intermediate caseload in contrast to low caseload Centers (*p* = 0.01).

## Results: Literature review

### Study selection process

Using a combination of keywords, MeSH and Emtree hierarchical terms, the investigators found 537 potentially relevant articles, saved in a unique Pubmed (.nbib) file, which was then imported into Endnote for to identify possible duplicates. After removal of duplicates and paper published before 2000, 158 studies were deleted. Overall, 379 studies were screened by title, and subsequently by abstract, leading to exclude 201 more studies (Cohen’s k coefficient = 0.91).

Studies candidates for full-text reading were 178, of which 152 were removed according to exclusion criteria (Cohen’s k coefficient = 0.97).

The remaining 26 studies underwent a further selection process by strict application of inclusion criteria. In closing, 18 retrospective comparative studies were selected for the meta-analysis including 17,199 patients operated for intracranial meningiomas. A total of 4164 patients were included in the *Elderly-Meningioma* group (MEP, Experimental Group), and 13035 in the *Young-Meningioma* group (YMG, Control Group).

**Fig. 1:**
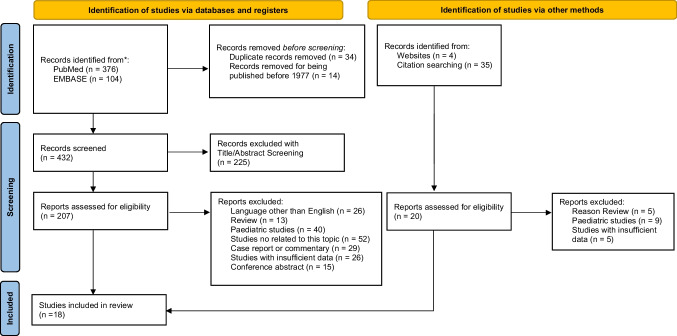
PRIMA flowchart. *The following filters were applied directly to the main search query: only English, no reviews and case reports. Only studies with a clinical application in patients were eligible, Study range was set between 2000 and 2023. ** The first number refers to records identified via keywords, the second refers to records identified via MeSH. ***The first number refers to records identified via keywords, the second refers to records identified via Emtree

**Fig. 2:**
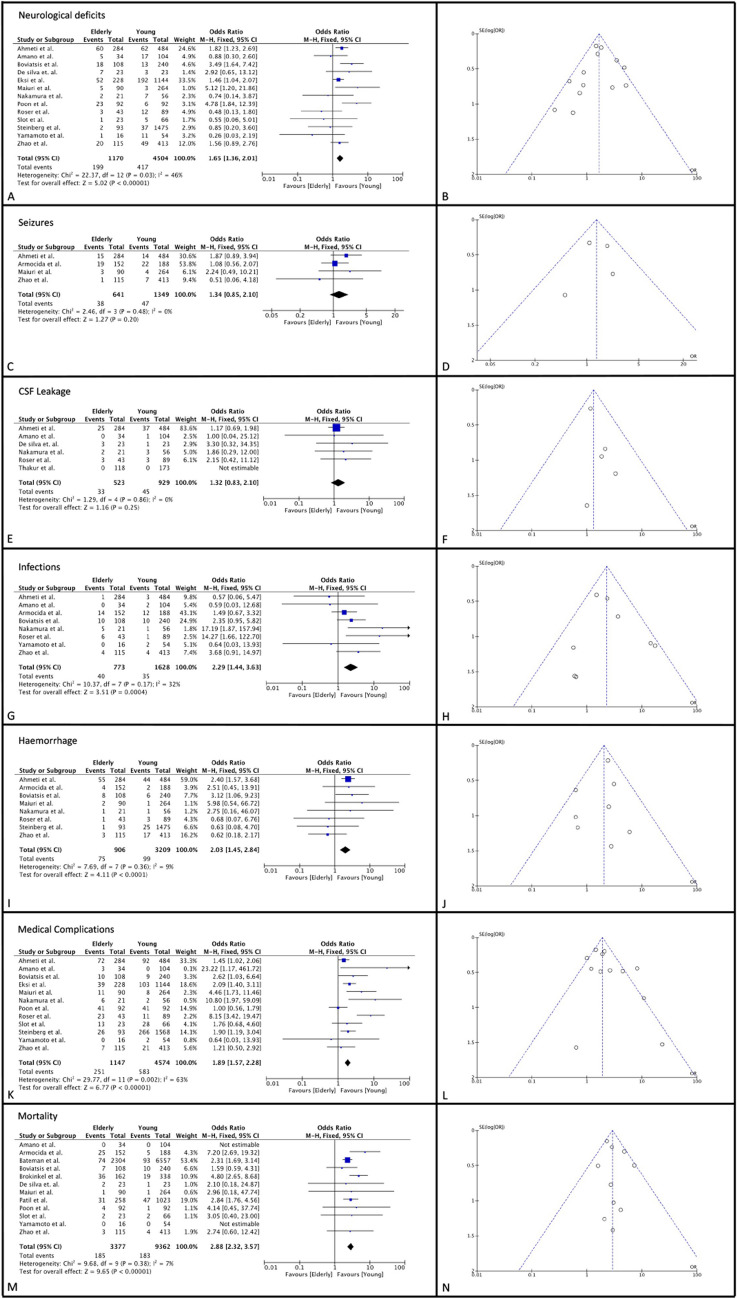
Forest plot and funnel plot for neurological deficits (**A**, **B**), seizure onset (**C**, **D**), CSF leakage (**E**, **F**), infections (**G**, **H**), Hemorrhage (**I**, **J**), medical complications (**K**, **L**) and mortality (**M**, **N**)

The selection study process is summarized in Fig. 1 following PRISMA guidelines. All the findings of this systematic review are summarized in Table 3. Data regarding the comparative analysis of morbidity and mortality between MEP and YMG are shown in Fig. 2.

**Table 3:** Summary of the retrospective comparative studies included in the meta-analysis

Authors	N° of Total Cases	Young Cases	Elderly Cases	Elderly cut-off value used	Scale of Clinical Risk	P.O Neurological deficits *Y-Group*	P.O Neurological deficits E*-Group*	P.O Medical Complications *Y-Group*	P.O Medical Complications E*-Group*	P.O Seizure Onset *Y-Group*	P.O Seizure Onset *E-Group*	Overall Mortality *Y-Group*	Overall Mortality *E-Group*
Nakamura et al. 2005 [17]	77	56	21	70	ASA, KPS	7 (12,5%)	2 (9,5%)	9 (16%)	8 (38,1%)	NA	NA	NA	NA
Bateman et al. 2005 [12]	8861	6557	2304	70	CCI	NA	NA	1086 (16,3%)	1222 (60%)	NA	NA	93 (1,4%)	74 (3,6%)
Boviatsis et al. 2007 [6]	348	240	108	65	ASA	13 (5,4)	18 (16,6%)	22 (9,1)	28 (25,9%)	NA	NA	10 (4,1%)	7 (6,4%)
Roser et al. 2007 [19]	132	89	43	70	ASA	12 (13,4%)	3 (6,9%)	19 (21,3%)	35 (81,4%)	NA	NA	NA	NA
Patil et al. 2010 [18]	1281	1023	258	70	ASA	NA	NA	134 (13,1)	77 (29,8%)	NA	NA	47 (4,5%)	31 (12%)
Poon et al. 2013 [7]	184	92	92	65	ASA	6 (6,5%)	23(25%)	47 (51%)	64 (69,5%)	NA	NA	1 (1,1%)	4 (4,3%)
Brokinkel et al. 2017 [13]	500	338	162	65	NONE	NA	NA	NA	NA	NA	NA	19 (5,6%)	36 (22,2%)
Yamamoto et al. 2017 [21]	70	54	16	65	GRS, skale, GSS	1 (1,8%)	11(68,7%)	13 (24%)	1 (6,2%)	NA	NA	0 (0%)	0 (0%)
Steinberg et al. 2017 [5]	1568	1475	93	80	ASA	37 (2,5%)	2 (2,1%)	303 (20,5%)	28 (30,1%)	NA	NA	NA	NA
de silva et al. 2018 [14]	46	23	23	65	skale	3 (13%)	7 (30%)	NA	NA	NA	NA	1 (4,3%)	2 (8,7%)
Amano et al. 2018 [10]	138	104	34	65	ASA	17 (16,3%)	5 (14,7%)	5 (4,8%)	20(58,8%)	NA	NA	0 (0%)	0 (0%)
Zhao et al. 2018 [22]	528	413	115	65	GOS, KPS	49 (11,8%)	20 (17,3%)	70 (16,9%)	27 (23,4%)	7 (1.69%)	1 (0.87%)	4 (0,9%)	3 (2,6%)
Slot et al. 2018 [8]	89	66	23	65	GOS, KPS, ASA	1 (1,5%)	5 (21,7%)	33 (50%)	14 (60,8%)	NA	NA	2 (3%)	2 (8,7%)
Eksi et al. 2019 [15]	1372	1144	228	65	ASA	192 (16,7%)	52 (22,8%)	295 (25,7%)	91 (39,9%)	NA	NA	NA	NA
Thakur et al.2020 [20]	291	173	118	65	ASA, KPS	NA	NA	16 (7,5%)	11 (9,3%)	NA	NA	NA	NA
Ahmeti et al. 2021 [9]	768	484	284	65	ASA, KPS	62 (12,8%)	60 (21,1%)	154 (31,8%)	132 (46,4%)	14 (2.89%)	15 (5.28%)	NA	NA
Armocida et al. 2022 [11]	340	188	152	65	ASA, KPS	NA	NA	NA	NA	22 (6.47%)	19 (12.5%)	5 (1,4%)	25 (16,4%)
Maiuri et al. 2023 [16]	354	264	90	70	ASA, CCI	3 (1,1%)	5 (5,5%)	11 (4,1%)	16 (17,7%)	4 (1.52%)	3 (3.33%)	1 (0,3%)	1 (1,1%)

### Bias assessment

The analyzed studies encompassed only observational retrospective studies. The bias assessment was conducted across all 7 domains according to Cochrane guidelines showing a low risk of bias in 20 papers, moderate in 2 papers, serious in 3 papers and critical in 1 paper. Bias evaluation data are summarized in Figure S1-S2. The funnel plot analysis showed the lack of a significant heterogeneity (> 50%) due to publication bias for the quantitative synthesis of all analyzed outcomes, except in the cases of medical complications for which a heterogeneity of 63% was observed.

### Neurological complications’ rate

A total of 16947 patients from 18 studies were included [5–22]. A total of 4164 belonged to MEP, whereas 12783 to the YMG. In the pooled meta-analysis, the MEP had a significantly increased risk of new postoperative permanent neurological deficits as compared to YMG (OR = 1.65,95%CI [1.36- 2.01], (*p* < 0.00001).There was a moderate heterogeneity, which however was considered not significant (I^2^ = 46). Four studies [9, 11, 16, 22] including 1990 patients provided data regarding postoperative new seizures in the two groups (641 in MEP and 1349 in YMG). The pooled analyses showed that patients in the MEP had a slightly higher risk of developing seizures in the postoperative period (OR = 1.34,95% CI [0.85–2.10], *p* = 0.20). The analysis showed no heterogeneity (I^2^ = 0%, *p* = 0.008).

### Surgical complications’ rate

The risk of developing postoperative CSF Leakage was analyzed in 1452 patients from 5 studies [9, 10, 14, 17, 19, 20]: it was similar in the two study groups with no statistical differences (*p* = 0.25). Conversely, the pooled analysis showed the MEP had a significant higher risk of postoperative infections (OR 2.29 CI [1.44–3.63], *p* = 0.0004; data from 8 studies [5, 6, 9, 11, 16, 17, 19, 22] and of hemorrhagic complications (OR 2.03 CI [1.45–2.84], *p* < 0.0001; data from 9 investigations [5, 6, 6, 9, 11, 14, 17, 19, 22].The heterogeneity was considered not significant and accounted for 32% and 7%, respectively.

### Medical complications’ rate and mortality risk

The overall medical complication rate was reported in 12 studies [5–10, 15–17, 19] including 5721 patients (1147 in the MEP vs. 4574 in the YMG). After quantitative analysis, the MEP showed a significantly increased risk for developing postoperative medical complications in comparison to YMG (OR = 1.89, 95%CI [1.57.- 2.28], (*p* < 00001). However, the analysis was affected by a significant heterogeneity (63%), probably due to the inclusion of studies including a small number of patients and, therefore, of observed infections.

Data on cumulative mortality rates have been documented in 12 papers [6–8, 10–14, 16, 18, 21, 22]. The reported time interval for perioperative mortality was not uniform among different studies and ranged from 30 to 90 days. The pooled analyses included 12,739 patient (3377 in EGM and 9362 in YMG) and documented a significant higher perioperative surgical-related mortality in the MEP vs. the YMG (OR = 2.88, 95%CI [2.32–3.57], *p* < 0.00001). The observed heterogeneity was not significant (I^2^ = 7%).

## Discussion

Despite a growing literature in favor of surgical management for symptomatic or growing MEP, the results in terms of morbidity and mortality often conflict with comparative studies between MEP and YMG [5–22].

This investigation aimed to provide a comprehensive overview of the current clinical practices and attitudes of Italian neurosurgeons regarding the MEP management, comparing the findings to current literature. To address the variability in study design, sample size, and endpoint analysis, the study utilized a meta-analysis approach to improve recommendations for MEP management.

1. **Elderly definition and risk scale**
  Due to the rising life expectancy and global population aging, there is an increasing number of elderly patients seeking surgical procedures, including meningioma patients [24, 26]. It is thus crucial to prioritize safety and carefully balance between surgery and neurological function preservation in this age group, given their reduced resilience and shorter life expectancy compared to younger patients [23, 24, 26]. Despite numerous scoring systems being suggested to identify patients at risk or who might benefit from surgery, none of them have gained widespread adoption, mainly for the insufficient evidence to support the routine use of various scoring systems proposed for preoperative risk assessment in MEP [26].
  The main 4 predictive grading systems in MEP demonstrated correlations with mortality but are lacking in including important clinical-radiological variables. The Clinical Radiological Grading System (CRGS), the Geriatric Grading System (GSS) and the Charlson Comorbidity Scale (CCS) do not take into account patient sex, despite recent large series identifying it as a prognostic factor. Conversely, the SKALE system does not include tumor size or preoperative neurological deficits in its assessment. Additionally, while CRGS, SKALE, GSS, and CCS all consider comorbidities in their evaluations, the latter does not incorporate the radiological features of the tumor [27].
  Another important bias in scoring predictive value could rely on the high heterogeneity among studies in the cut-off selection used to define a patient as elderly.
  Despite the heterogeneity of the cut-off used in the current literature to identify the elderly patients(REF), 66.7% of responders identify patients over 70 years of age as elderly.
  In recent investigations the age-cutoff to define “elderly” patients was mostly set at 70–75 years of age [5–22], opening a separate parenthesis regarding intrinsic fragility consideration for cases over.
  eighty-year-old. A close collaboration between medical teams and surgeons should provide novel scoring systems more reliable in predicting postoperative in MEP surgery [26–28].
2. **Incidental meningioma**
  Incidental findings of meningioma are gradually increasing mainly for the widespread of neuroradiological exams performed for other medical reasons, such as minor head injury. A recent meta-regression analysis showed that the prevalence of incidental meningioma was significantly higher in elderly individuals [29]. Although majority of incidental meningiomas are known to be indolent, the selection of patients who are suitable for treatment is still controversial [30]. Given the slow growth of most intracranial meningiomas and the potential slower growth rate in older patients compared to younger ones, waiting and see approach as a primary treatment option for incidental asymptomatic meningioma should be recommended, especially for SBMs in the elderly [3–5, 7, 11].
  In this survey all respondents agree to state the option of surveillance at initial presentation for asymptomatic incidental cases. This approach is supported by growing evidence suggesting that most small, asymptomatic meningiomas without edema rarely enlarge [3].
  For those cases showing a volumetric MRI progression at follow-up surgery is chosen as preferential treatment option from more than half of Centers, while SRS is selected from 11.8% of responders. No guidelines are available for this clinical setting. Considering that Atypical meningiomas may grow exponentially, whereas benign meningiomas exhibit exponential-linear, or no growth, the analysis of tumor growing pattern should be included in the workflow to improve the treatment selection [28, 30–32].
  The risk of meningioma growth could be assessed on clinical and imaging factors in order to define the need for follow-up stratified by patient age, comorbidity and performance status. A proposed prognostic model to personalize monitoring regimes observed little benefit to rigorous monitoring in low risk MEP with comorbidities. An accurate stratification of patients and risk of meningioma growth may allow to reduce both health care costs and patients’ anxiety for uncertainty of the need for future treatment [33].
3. **Surgery**
  Surgical management of MEP has become a rising challenge as for the overall increasing life expectancy and improving quality of life in elderly patients. Although there are no randomized controlled trials directly comparing the advantages of surgical removal to observation or radiation in MEP, surgical resection remains the gold standard of treatment for large, symptomatic lesions, and rapidly growing tumors under surveillance [5–22, 24, 26–28, 31–35]. In this survey, most Centers (76.4%) stated that age per se is not a factor influencing the surgical decision, being in line with the common attitude reported in recent literature.
  The lack of widespread use of objective measurement tools, extensive variability in the definition of "elderly age", differences in the grading systems to assess comorbidity in the cohorts make the investigations heterogenous and difficult to compare [5, 7, 9, 18, 24, 26, 27, 30–34]. In addition, the emerging predictive role of frailty index as surrogates of biological reserve against stressors, a priori selection, in surgical series, of patients in stable medical conditions poses the critical issue of selection bias in the comparative analysis and shed the light on the need to considered the impact of novel risk factor on surgical outcome [24].
  The existing literature on morbidity and mortality following craniotomy for meningioma in the elderly is conflicting [7, 8, 13, 18].
  Many studies describe advanced age as a risk factor for morbidity and mortality in the perioperative period. However, others have concluded that there is no statistically significant relationship between these variables, though many of these investigations have small sample sizes, and use different age cut off [8, 13, 18].
  Overall, in this investigation, the pooled meta-analysis pointed out that Surgical management of MEP had a significantly increased risk of new postoperative permanent neurological deficits (p < 0.00001), higher risk of postoperative infections (p = 0.0004) and of hemorrhagic complications, increased risk for developing postoperative medical complications (p < 00001), and a significant higher perioperative surgical-related mortality (p < 00001), in comparison to YMG.
  Despite the feasibility and success of surgery in controlling symptoms and managing the disease, it is essential to take these results into consideration during the patient counseling and decision-making process.
  Moreover, prolonged operative durations can pose risks for complications and mortality in the elderly. Therefore, employing a strategy involving subtotal resection may be beneficial in reducing operative time, especially given that GTR may not necessarily offer a survival advantage in older individuals, mainly in those cases with challenging skull base lesions. STR can effectively alleviate symptoms, and significant recurrence within the patient's remaining lifetime is unlikely in very elderly cases.
  Meningioma interventions are typically not urgent, allowing for adequate medical optimization of elderly patients. Despite the recognition of the importance of multidisciplinary discussion, this attitude is still not widespread in our country, strictly correlated with the Center caseload.
  The great versatility of the endoscopic endonasal corridor has allowed it to expand the horizons from pituitary lesions to several base tumors, encompassing also meningiomas arising at the midline skull base. Along with surgical experience, selected meningiomas, mostly those originating from the tuberculum, the planum sphenoidale, have been considered amenable to transnasal endoscopic resection [35–37].
  The expanding surgical capabilities of adopting such a technique has led to achieve satisfactory outcomes, above all in terms of visual functions [38, 39].
  The results of the present survey confirm the actual trends: most of the centers having experienced with endonasal endoscopic approach consider it a viable solution when dealing with midline skullbase meningiomas,—mostly at midline. Endoscopic endonasal approach offers a wide exposure enough to manage tumor removal maneuver, while limiting brain retraction and manipulation of critical neurovascular structures. Above all, it provides a more direct and early access to the tumor with the possibility of immediately controlling the blood supply: this results in a lesser bleeding mass, which is better dissected and removed, thus, the operative timing and surgical morbidity can be reduced.
  As final surgical consideration, managing a meningioma with bone infiltration requires removal of the tumor-infiltrated bone and subsequent cranioplasty, which plays a pivotal role in restoring the functionality and cranial vault's anatomical integrity. This survey highlights that for convexity meningiomas with notable hyperostosis, almost all Centers opt to perform the cranioplasty procedure concurrently with the surgical meningioma resection. However, the choice of materials for this procedure varies widely among centers, despite the growing interest in porous hydroxyapatite (PHA) for its potential in bone integration [40]. Performing cranioplasty at the same time as resection can lead, however, to longer surgery, which might be problematic for elderly patients.
4. **Radiosurgery and stereotactic radiotherapy**
  Over the last two decades, stereotactic radiosurgery (SRS) and stereotactic radiotherapy (SRT), especially in hypo-fractionated fashion (HFSRT), have become alternative strategies in multimodal management of meningioma whether as primary treatment or as an adjuvant therapy, especially in fragile patients or for complex skull-base lesions [4, 41].
  SRS and SRT are currently available respectively in 5.9% and 74.5% of centers participating to the survey, reflecting the emerging role of these techniques.
  SRS and SRT are typically employed for meningiomas in challenging anatomical locations, for small lesions, or for patients experiencing tumor recurrence following incomplete resections.
  The efficacy of these methods in limiting tumor growth has been widely established, despite the ongoing debates concerning the optimal timing, prescription doses, and fractionation of delivery [42–44]. Progression free survival rates varied from 78.0% – 98.9% and 53.1% – 97.2% at 5 and 10 years, respectively; overall symptom control was 92.3%, and overall toxicity was 8.1%. The estimated disease control rate ranged from 87.0% to 100.0% at 5 years and from 67.0% to 100.0% at 10 years [41].
  Only a few studies, however, took into account the roles of SRS and SRT in older patients, demonstrating good tumor control with low toxicity rates in MEP, despite variations in prescription doses, fractionation of delivery (single or hypo-fractionation), and technology used (LINAC device and Gamma Knife) [41–45].
  Rueß and colleagues have shown that patients' co-morbidities do not impact the efficacy or, more importantly, the toxicity of the treatment, which is in contrast to surgery, where this is consistently a concern [45]. According to a recent meta-analysis of MEP, postoperative mortality and pre-existing medical conditions are commonly related [46].
  Similarly, Eksi et al. highlighted that comorbidities strongly predict postsurgical neurological complications [15]. These findings emphasize the importance of careful patient selection and thorough case evaluation within a multidisciplinary team. This integrative management approach has certainly to be strengthened in the Italian neurosurgical setting, considering that it is currently adopted only from 37.3% of centers.
  SRS approach was selected as the first option in selected cases of MEP (lesions with a maximum diameter < 3 cm) by 76.4% of responders, in residual management for 66.7%, and in patients with high preoperative risk in 39.2%).
  Elderly patients have often larger tumors compared with the tumors of younger patients then Zhao et al. concluded that “rather than achieving total resection, conservative and safety preferential treatment strategies should be regarded as a higher priority for better quality of life” [22]. A presurgical evaluation of adaptive surgery followed by SRS or SRT, in MEP with severe comorbidities or complex skull base lesions, is still poorly developed in the current literature, despite its valuable clinical rationale.
5. **Perioperative and post-operative management**
  In the domain of the preoperative and postoperative management of MEP three topics has been considered: 1. The role of Preoperative Digital Subtraction Angiography (DSA) and embolization; 2. Venous thromboembolism prophylaxis; 3. The postoperative adjuvant treatment.
  5.1. **Preoperative DSA and embolization**
    The hypervascularity, size, and deep location at the skull base meningioma can make them surgically challenging. Preoperative embolization may aid in decreasing intraoperative blood loss and transfusion requirements, although the conflicting results have precluded the integration of the procedure into standard clinical practice. In this survey 84% of responders stated the preoperative use of endovascular embolization in selected cases, i.e. deep lesions with vascular encasement or giant hyper-vascularized lesions. Currently, there are no randomized clinical trials or extensive comparative studies that definitively establish preoperative embolization as a standard treatment for meningiomas. Therefore, the decision to perform embolization before surgery must be personalized for each patient, considering factors such as the expected blood loss and the challenges in securing the vascular supply during the operation [47].
  5.2. **Venous thromboembolism prophylaxis**
    Meningioma resection poses a 3–4% risk of VTE. Preventive measures, though extensively studied, remain controversial. In this survey, VTE prophylaxis approach is in line with the literature data: 84.6% of neurosurgeons reported to start early chemoprophylaxis within 24 h and, 73.1% prescribe a CT scan before starting the therapy. Complementary non-chemical conservative methods such as intermittent venous compression or using stockings are adopted with wide heterogeneity.
    A combination of chemoprophylaxis and mechanical prophylaxis is recommended after meningioma surgery. A meta-analysis by Khan et al. found that chemoprophylaxis is beneficial in preventing VTE without significantly increasing bleeding complications [48]. Patients who developed VTE were more likely to have received their first postoperative dose later, as demonstrated by Tan et al. [49]. Additionally, non-chemical methods have been shown to be safe and effective in preventing VTE in MEP [50].
  5.3. **Postoperative adjuvant treatments**
    Treatment concepts combining surgery and radiosurgery or fractionated RT are increasingly used, although there remain controversies regarding timing, type, and dosing of the various RT approaches.
    For WHO 1 meningiomas with incomplete removal, most respondents (82.4%) favored a "wait and scan" strategy with regular MRI follow-ups, as rapid progression in this clinical scenario is rare. However, RT or SRS can be considered an adjuvant or salvage therapy for recurrent WHO 1 tumors or as adjuvant treatment for complex cases, such as cavernous sinus or SBM [4, 51]. In case of STR of WHO2 meningioma adjuvant RT is considered standard practice according to EANO guidelines. Otherwise, the indication for complementary treatments remains a matter of debate in case of GTR [4, 51].
    RT may be effective in local control and progression-free survival, but currently no strong evidence supports a benefit in overall survival. In elderly, a higher risk of recurrence after completely resected WHO2 meningiomas is reported, but evidence supporting the role of RT is still lacking [3, 4, 51].
    WHO3 meningiomas exhibit local aggressiveness and generally result in poor prognosis, particularly among elderly patients. The standard recommendation is thus to consider adjuvant RT, which seems to offer a substantial survival advantage for most patients [3, 4].
    Postoperative adjuvant RT, particle or photon-based, for WHO 3 meningioma was thus selected by all responders to the survey.
    Nevertheless, in the elderly population, the survival benefit of adjuvant RT for WHO3 meningiomas may be restricted to those who have undergone STR, as studies indicate that RT does not confer a survival advantage following GTR [3].
    In closing, the RT effectiveness in enhancing survival for non-malignant meningiomas remains inconclusive. Some data hints at the possibility of decreased survival among elderly patients who receive postoperative RT after complete tumor removal, but this could be influenced by patient-specific factors [3, 4, 27]. As a result, decisions regarding the use of adjuvant therapies such as SRS or RT should be tailored to individual cases, considering factors such as symptoms, tumor subtype, patient preferences, care goals, performance status, and the extent of initial surgical resection. This underscores the importance of case-specific discussions within multidisciplinary medical teams.

### Limitations and strengths

Although the survey was distributed across various neurosurgical departments in Italy, participation remains self-selective. This could have resulted in a non-representative group of responders, which could be biased towards treatments options. Despite these limitations considering the higher percentage of responders reached, we believe this study provides valuable insight into the current options for surgical management of MEP among neurosurgeons in Italy.

The quality of the meta-analysis results is constrained due to the absence of prospective studies on the subject. Extensive prospective investigations are needed to compare outcomes between MEP and YMG adopting a standardization of patient selection, surgical methods, complication reports, and accurate documentation about timing and reasons of mortality.

Furthermore, the included studies span nearly 20 years, during which brain tumor surgery and anesthesiologist techniques have evolved, but the extensive number of studies and robust statistical analyses enhance the study's reliability.

## Conclusions

Meningioma elderly patients are becoming more common in clinical practice with a growing number of asymptomatic cases. Vigilant monitoring should be included among the valid options if the lesion is asymptomatic or mild symptomatic and whenever there is radiological stability. Surgical treatment is the primary approach for symptomatic cases, yielding better results in carefully selected patients. Age alone should not deter intervention in elderly patients with good initial function and few medical issues. When surgery isn't an option, SRS is increasingly considered. However, a meta-analysis shows higher postoperative risks for elderly patients, highlighting the need for better outcome predictors. The survey's findings emphasize the importance of multidisciplinary evaluation in decision-making process and the need for standardized guidelines to develop national recommendations for clinical practice, education, and research.

## Supplementary Information

Below is the link to the electronic supplementary material.

Supplementary file1 (PNG 165 KB) Figure S1: Risk of bias estimation for the selected papers according to the robvis tool

Supplementary file2 (PNG 1588 KB) Figure S2: Risk of bias estimation for the selected papers according to the robvis tool

Supplementary file3 (XLSX 20 KB) Figure S3: SINch NeuroOncology Study Group

## Data Availability

No datasets were generated or analysed during the current study.
