# Supplementary figures and images for: From data to practice: brain meningioma treatment in elderly patients – a survey of the Italian Society of Neurosurgery (SINch®) and systematic review and meta-analysis
Source: Neurosurg Rev. 2024 Jul 31;47(1):373. doi: 10.1007/s10143-024-02524-8 (PMC11291526; doi:10.1007/s10143-024-02524-8)

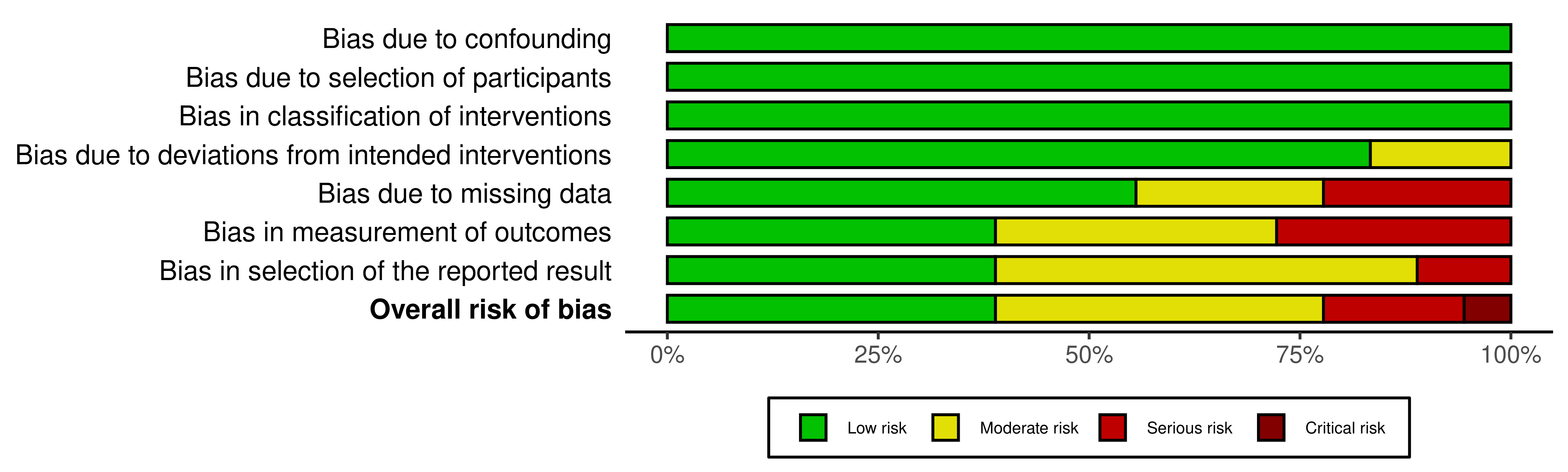

Supplement: Supplementary file 1 — Supplementary file1 (PNG 165 KB) Figure S1: Risk of bias estimation for the selected papers according to the robvis tool [file 10143_2024_2524_MOESM1_ESM.png]

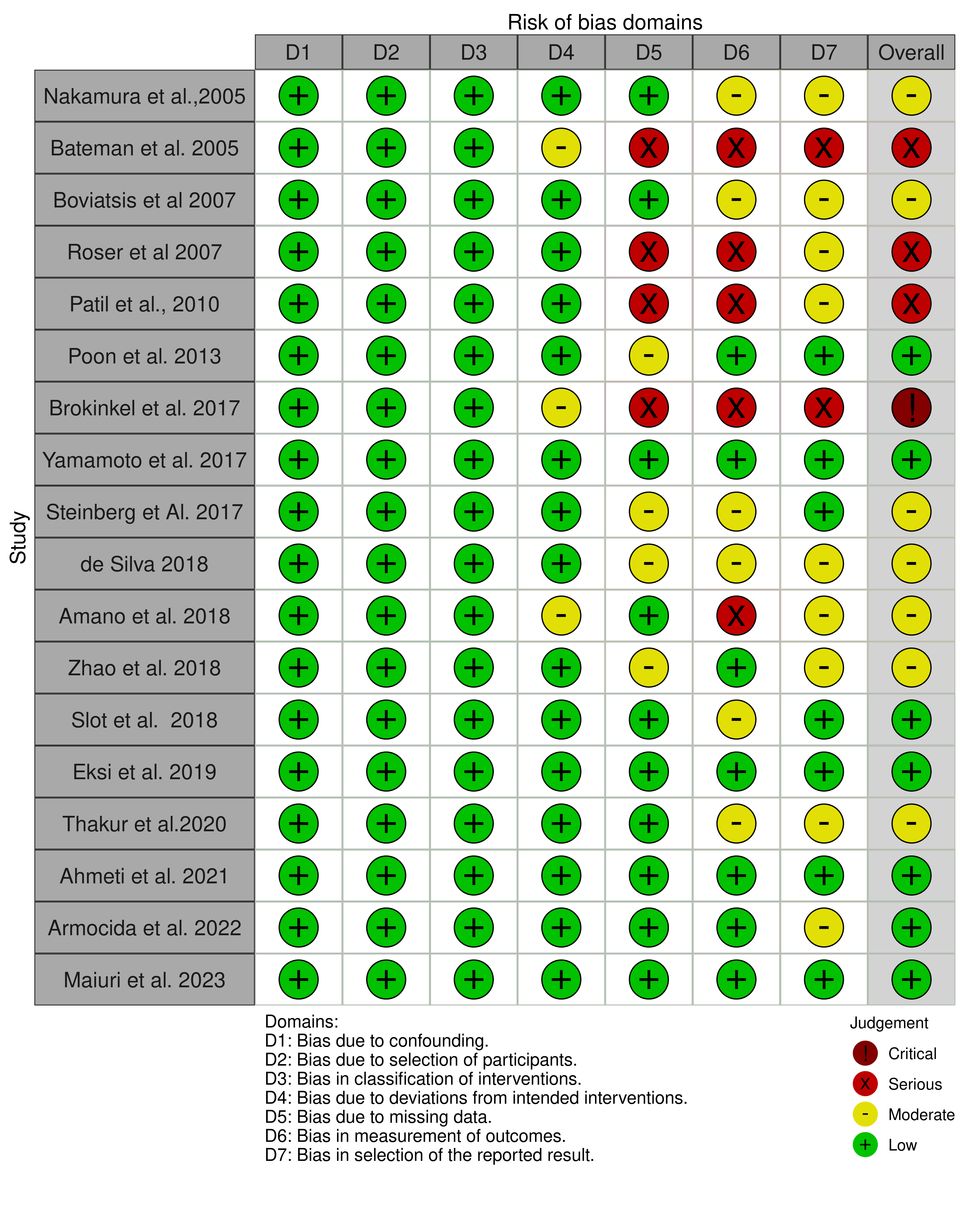

Supplement: Supplementary file 2 — Supplementary file2 (PNG 1588 KB) Figure S2: Risk of bias estimation for the selected papers according to the robvis tool [file 10143_2024_2524_MOESM2_ESM.png]
